# Editorial: Pharmacoepidemiology and pharmacovigilance post-marketing drug safety studies

**DOI:** 10.3389/fphar.2024.1473052

**Published:** 2024-08-20

**Authors:** Zakir Khan, Yusuf Karatas, Ahmet Akici, Maria Auxiliadora Parreiras Martins, Nafees Ahmad

**Affiliations:** ^1^ Riphah Institute of Pharmaceutical Sciences, Riphah International University, Gulberg Greens Campus, Islamabad, Pakistan; ^2^ Department of Medical Pharmacology, Faculty of Medicine, Çukurova University, Adana, Türkiye; ^3^ Department of Medical Pharmacology, School of Medicine, Marmara University, Maltepe, Türkiye; ^4^ School of Pharmacy, Universidade Federal de Minas Gerais (UFMG), Minas Gerais, Brazil; ^5^ Department of Pharmacy Practice, Faculty of Pharmacy and Health Sciences, University of Balochistan, Quetta, Pakistan

**Keywords:** pharmacovigilance, drug, patient safety, real world data (RWD), adverse events

## Introduction

Pharmacoepidemiology defined as “the study of the usage and effects of drugs in a large group of people” enables the cost-effective inclusion of significantly larger number of patients compared to pre-marketing studies, leading to a more accurate measurement of both the adverse and beneficial effects of drugs. Pharmacoepidemiological studies provide better quantification of the incidence of known adverse and beneficial effects in patients not enrolled in clinical trials, and those with multiple diseases and taking concurrent medication. These studies also assess the relative effectiveness and safety of drugs used for the same condition. Additionally, they provide information not obtainable from pre-marketing studies, such as insight into rare and delayed effects, pattern of drug utilization, effects of overdose, economic implications of drug use, and reassurance regarding drug safety (Strom, 2019; Crescioli et al., 2022). Whereas, pharmacovigilance defined as “the science and practices relating to the identification, assessment, understanding, and prevention of adverse drug events or any other potential drug-related problems” (WHO, 2002). It plays an important role in post-marketing drug safety studies. It encompasses detecting, assessing and reporting adverse drug reactions (ADRs), signal detection, elucidation of pharmacological and toxicological properties of drugs, identifying high risk patients, drug interactions, ADRs risk management, post-marketing surveillance, and taking regulatory actions when safety concerns arise (Raj et al., 2019; Trifirò and Crisafulli, 2022).

The full implementation of pharmacovigilance practices is one of the prerequisites for the rational use of drugs. Pharmacoepidemiology provides vital methodological support in achieving and maintaining this pharmacovigilance contribution in the periods before, during and after the use of drugs (Akici and Oktay, 2007; Gulmez et al., 2020). Pharmacoepidemiology and pharmacovigilance work in synergy (Bérard, 2021; Lavertu et al., 2021; Crescioli et al., 2022). These studies are crucial for augmenting pre-marketing evidence, providing insights into the risk-benefit profile of drugs as well as its use in larger patient populations. They hold significant potential to contribute to post-marketing drug safety assessments. Utilizing real-world data sources, including spontaneous reporting system databases like the World Health Organization (WHO) global pharmacovigilance database (VigiBase), the FDA Adverse Event Reporting System (FAERS), the EudraVigilance database of European Medication Agency, as well as claims databases, electronic healthcare records, and drug/disease registers, pharmacovigilance and pharmacoepidemiologic studies continuously monitor approved and marketed drugs. This ongoing monitoring process is crucial for the timely detection of new ADRs (Bérard, 2021; Crisafulli et al., 2023).

This Research Topic included 16 articles with various study approaches. These included disproportionality analysis of antiarrhythmic drugs (AADs) associated with cardiac arrhythmias by Wang et al. Additionally, Wei et al. investigated differences in adverse events among methylphenidate, atomoxetine and amphetamine, adverse events associated with molnupiravir by Liang et al. and post-marketing safety surveillance of sacituzumab govitecan were examined by utilizing FDA FAERS database in a study conducted by Liu et al. Moreover, Liu et al. also examined the pharmacovigilance and clinical characteristics of heparin-induced thrombocytopenia caused by low-molecular-weight heparin. Giner-Soriano et al. explored the information on the effectiveness and safety of oral anti-coagulants for the prevention of stroke in non-valvular atrial fibrillation was gathered from the electronic health records of Primary Healthcare. Data mining of the WHO global pharmacovigilance database (VigiBase) was conducted Sharif et al. to examine sex -specific safety response to dual Interleukin 4 (IL-4) and Interleukin 13 (IL-13) blockade by dupilumab. Prevost et al. analyzed the real-world cases of neurocognitive impairment with endocrine therapies and cycline-dependent-kinase-4/6 inhibitors (iCDK4/6s) in breast cancer were analyzed by utilizing WHO VigiBase. An opinion based article by Luthra and Toklu within this Research Topic underscored the importance of reporting safety concerns and adverse events associated with nutraceuticals. The authors advocated for the incorporation of pharmacovigilance into health programs curricula, public awareness initiatives and future post-marketing drug safety studies, specifically emphasizing the need for nutrivigilance to enhance the overall pharmacovigilance system.

A study conducted in Korea by Kim et al. compared the antidepressant ADR signal profiles between data from the national health insurance claim and the Korea Adverse Event Reporting System highlighted the importance of integrating data from various sources, providing significant regulatory insights and broadening the scope of pharmacovigilance. Zou et al. examines the FDA’s FAERS database in relation to mepolizumab side effects including 18,040 reports of adverse events linked to mepolizumab. This study indicated that these details will be very helpful in putting the medication to use practically in clinical settings.

A study conducted by Xia et al. observed the possible correlation between pericarditis and biological disease-modifying antirheumatic medications (bDMARDs) as well as the clinical features of ankylosing spondylitis (AS). This study reported 1,874 reports of pericarditis caused by bDMARDs (11.3% of which were fatal). This study encouraged additional studies to understand the underlying mechanisms and uncover patient-related susceptibility factors, therefore promoting earlier diagnosis and safer prescribing of bDMARDs. While, Sun et al. examined the AE signals using four anti- Calcitonin gene-related peptide antibodies (CGRP) monoclonal antibodies (mAbs) including erenumab, galcanezumab, fremanezumab and eptinezumab in the FDA FAERS database to investigate the post-marketing safety profile of these drugs. The number of reports obtained from FDA FAERS database for erenumab, galcanezumab, fremanezumab and eptinezumab were 38,515, 19,485, 5,332, and 2,460, respectively. This study has detected new adverse events (AEs) such as menstruation disorders, Raynaud’s phenomenon, weight gain, throat tightness and oral paraesthesia that were not mentioned in the drug leaflets but appeared simultaneously with multiple drugs. These findings contribute to our understanding of anti-CGRP mAb safety in clinical settings and providing valuable information regarding the clinical selection of drugs.

Furthermore, Lu et al. study included in this Research Topic uses the FDA FAERS database to examine human serum albumin (HSA) adverse event signals for the safe therapeutic usage of this medication. This study used the Medicines and Healthcare Products Regulatory Agency (MHRA), the Bayesian confidence propagation neural network (BCPNN), and the reporting odds ratio (ROR) to clean and analyze adverse event reports for 76 quarters (Q) spanning Q1 2004 to Q4 2022. A total of 535 reports of adverse events were found using a combination of three techniques. These reports comprised 1,885 cases of adverse reactions; the most frequent ones involved respiratory, thoracic, mediastinal, general disorders and administration site problems. One notable new signal was the happening of transfusion-related acute lung injury. This study found that HSA increases the risk of transfusion-related acute lung injury. Moreover, adverse reactions such as hypertension, pulmonary oedema, paraesthesia, loss of consciousness and vomiting were more commonly observed in females. This study suggested more research to corroborate these findings. This study recommended further research to confirm these findings.

A retrospective study of drug-induced infusion reactions (IRs) conducted by Yin et al. in a hospital pharmacovigilance center during a 5-year period identified 505 cases of inpatient drug-induced IRs. About 105 cases (20.8%) were classified as severe IRs. According to this study, antibiotics and antineoplastic drugs were the main culprit drugs as per the local real-world data from hospital pharmacovigilance center. This study recommended a complete understanding regarding the clinical characteristics of IRs to enable active pharmacovigilance and the adoption of suitable preventive interventions for susceptible populations with risk factors. A recent study of Zhang et al. included 40,474 reports of oxaliplatin as the primary or secondary suspect drug revealed that few ADEs related to immune system disorders caused by oxaliplatin remain unrecognized, especially type II hypersensitivity, which displayed strong intensity signals as a pharmacovigilance signal. This study advocates for more observational real-world studies to better understand the prevalence of different AEs.

In conclusion, articles included in this Research Topic highlight the importance pharmacoepidemiology and pharmacovigilance studies in enhancing our understanding of drug utilization and safety issues in patients suffering from different diseases. Pharmacovigilance based studies are crucial in crisis situation such as the recent COVID-19 pandemic. It also advocates that real world data sources are instrumental in timely detection of ADRs and continued surveillance of marketed drugs throughout the duration of their use to ensure that their benefit to risk ratio are and remain in acceptable limits.
